# Improvement of Bonding Strength Between Polyphenylene Sulfide/Glass Fiber Composites and Epoxy via Atmospheric-Pressure Plasma Surface Treatment

**DOI:** 10.3390/polym17101344

**Published:** 2025-05-14

**Authors:** Hwan-Gi Do, Pyoung-Chan Lee, Beom-Gon Cho

**Affiliations:** 1 Department of Polymer Science and Engineering, Kumoh National Institute of Technology, 61 Daehak-ro, Gumi 39177, Gyeongbuk, Republic of Korea; 2025210703@kumoh.ac.kr; 2 Chassis & Materials Research Laboratory, Korea Automotive Technology Institute, 303 Pungse-ro, Pungse-myeon, Dongnam-gu, Cheonan-si 31214, Chungnam, Republic of Korea

**Keywords:** polyphenylene sulfide, glass fiber, composites, surface modification, atmospheric-pressure plasma, bonding strength

## Abstract

Polyphenylene sulfide (PPS) is becoming increasingly valuable in the electrical, electronic, and automotive industries. In particular, PPS composites reinforced with glass fiber (GF) have better dimensional stability and mechanical properties than conventional PPS materials and can be used in applications like electric vehicle capacitor housing. In the electric vehicle industry, the epoxy-molding process is essential for manufacturing capacitor housings, where the bonding strength between the PPS/GF composites and epoxy significantly affects the durability of the product. However, the inert surface characteristics of polymers like PPS limit their interaction with epoxy, decreasing the bonding strength. This study was aimed at enhancing the bonding strength between PPS/GF composites and epoxy by modifying the PPS surface using atmospheric-pressure plasma treatment. The surface modification resulted in increased surface roughness and the introduction of polar functional groups, which improved both mechanical interlocking and chemical affinity to the epoxy. Surface changes were analyzed using atomic force microscopy and scanning electron microscopy, and chemical characterization was conducted using X-ray photoelectron spectroscopy and Fourier-transform infrared spectroscopy. Surface energy was determined via contact angle measurements, and bonding strength was evaluated through single-lap shear tests. The results showed a 55% increase in surface energy and a 24.8% improvement in bonding strength due to the surface modification.

## 1. Introduction

Polyphenylene sulfide (PPS) is a super-engineered plastic known for its outstanding thermal stability, chemical resistance, and mechanical properties. In particular, PPS exhibits excellent dimensional stability and electrical insulation, making it widely used in electronics, aerospace, and automotive applications [1,2,3]. Compared with other thermoplastics like polyethylene terephthalate (PET) or polyamide (PA), PPS offers higher temperature resistance, flame retardancy, and long-term mechanical stability. In addition, compared with conventional thermosetting polymers such as epoxy and rigid polyurethane (RPU), PPS exhibits distinct advantages in terms of long-term performance and environmental resistance. Epoxy resins are prone to cracking and stress concentration under complex loading conditions, and their hydroxyl-rich molecular structure readily absorbs moisture, leading to hydrolysis, plasticization, and fiber/matrix debonding [4]. RPU is inherently brittle, and despite various reinforcement strategies, its mechanical limitations continue to hinder broader application [5]. In contrast, PPS offers excellent thermal resistance, chemical stability, mechanical performance, and recyclability due to its thermoplastic nature. Recent studies have demonstrated its potential as a reliable alternative in high-performance composite systems [1].

To improve its mechanical strength, PPS is often reinforced with fibers such as carbon fiber (CF) or glass fiber (GF). PPS-based composites reinforced with carbon fiber (CF) or glass fiber (GF) are lightweight and possess enhanced mechanical strength and dimensional stability compared with PPS [6,7,8]. Compared with CF, GF offers better cost-effectiveness and electrical insulation, which are desirable properties for electrical components such as capacitor housings. Therefore, GF-reinforced PPS (PPS/GF) is a promising material for applications such as capacitor housings in electric vehicles [9].

In electric vehicles, the fabrication of capacitor housings requires epoxy molding, where the bonding strength between the PPS/GF composites and the epoxy resin is a crucial factor influencing the final product’s performance. However, polymers like PPS inherently possess inert surfaces; therefore, formation of chemical bonds with adhesives is difficult. This issue arises owing to their non-polar nature, low surface energy, and lack of polar functional groups, all of which contribute to their weak bonding strength [10,11]. To overcome these limitations, surface treatments are essential for improving adhesion performance [12,13,14]. Several surface modification strategies have been explored, including chemical grafting, silane coupling, nanoparticle coating, laser pattering, and plasma treatment. Tutunchi et al. introduced a two-step chemical method combining acid oxidation and epichlorohydrin grafting, which significantly enhanced bonding strength with epoxy resin through strong chemical interactions. This method offers robust surface functionalization but involves multi-step wet chemistry and environmental concerns due to acid usage [15]. Nurazzi et al. reviewed silane-based treatments, showing that they are cost-effective and widely applicable for natural fibers, although they are sensitive to moisture and require precise control of reaction conditions [16]. Hwang et al. employed laser patterning micro-patterned CFRP surfaces, which improved adhesion via enhanced mechanical interlocking without significant damage to the fibers. However, the requirement for expensive equipment and the limited scalability remain key drawbacks [17]. Du et al. utilized silane coupling agents and carbon nanotube coatings to reinforce carbon fiber surfaces, achieving increased mechanical strength of the composites [18]. While effective, this hybrid approach involves complex surface preparation and material costs. In contrast, atmospheric-pressure plasma (APP) treatment is a promising surface modification technique that enables mass production via continuous processing, while preserving the intrinsic properties of materials by minimizing excessive energy exposure [19,20]. In addition, APP treatment is a dry, eco-friendly process, unlike wet chemical surface modification, making it suitable for heat-sensitive polymer materials. This plasma treatment was applied to PPS/GF composites and improved the bonding strength with epoxy through several mechanisms: (1) removal of organic surface contaminants; (2) etching-induced surface roughness enhancement, which increases the adhesive contact area; and (3) introduction of polar functional groups such as hydroxyl (–OH) and carboxyl (–COOH) groups, facilitating stronger chemical interactions with epoxy adhesives [21,22,23].

Although previous studies have examined plasma treatment on various thermoplastics [21,22,23], systematic analysis focusing on how plasma-treatment parameters (e.g., treatment speed) affect the adhesion of PPS/GF composites has not been sufficiently explored. Furthermore, few studies provide comprehensive correlation among surface chemistry, roughness, and mechanical bonding strength in PPS-based composites. In this study, we investigated the effects of APP treatment on the surface properties of PPS/GF composites and examined the influence of varying plasma-treatment speeds on the surface structure and bonding strength. The aim of this study is to enhance the adhesion performance in the epoxy-molding process of PPS/GF-based capacitor housings for electric vehicles through a simple, rapid, and effective atmospheric-pressure plasma treatment. In addition, the optimal plasma-treatment speed that is needed to maximize bonding strength is investigated. The chemical composition and functional group introduction were analyzed using X-ray photoelectron spectroscopy (XPS) and Fourier-transform infrared (FT-IR) spectroscopy. The changes in surface morphology and roughness were examined using atomic force microscopy (AFM) and scanning electron microscopy (SEM), whereas energy-dispersive spectroscopy (EDS) was used to evaluate the elemental composition of the etched surfaces. Contact angle measurements were conducted to assess the changes in surface energy, and the bonding strength was assessed using a single-lap shear test.

## 2. Materials and Methods

### 2.1. Preparation of PPS/GF Composites

The PPS compounds reinforced with 65 wt% short glass fiber were supplied by Toray Advanced Composites Corporation (Tokyo, Japan). The PPS matrix (TORELINA^TM^ series, Toray Advanced Composites Corporation, Tokyo, Japan) exhibits excellent thermal and mechanical properties, including a tensile strength of approximately 150 MPa, a tensile modulus of ~6.0 GPa, a flexural strength of ~496 MPa, and a heat deflection temperature (1.8 MPa) of approximately 237 °C. The glass fiber contributes additional strength, with a tensile strength of ~610 MPa and a tensile modulus of ~33.8 GPa. The resulting PPS/GF composites exhibit a composite density of approximately 1.73 g/cm^3^. The plate samples, with dimensions of 100 mm (width) × 100 mm (length) × 3 mm (thickness), were manufactured using an injection molding process at a barrel temperature of 300 °C and a mold temperature of 140 °C, injection pressure of 80 MPa, cooling time of 30 s, and mold holding time of 10 s [9]. Furthermore, the injected plate samples were cut to dimensions of 25 mm × 100 mm × 3 mm (width × length × thickness) for the single-lap shear test. A schematic illustration of the sample preparation is shown in Figure S1.

### 2.2. Atmospheric-Pressure Plasma Surface Treatment

**Figure 1:**
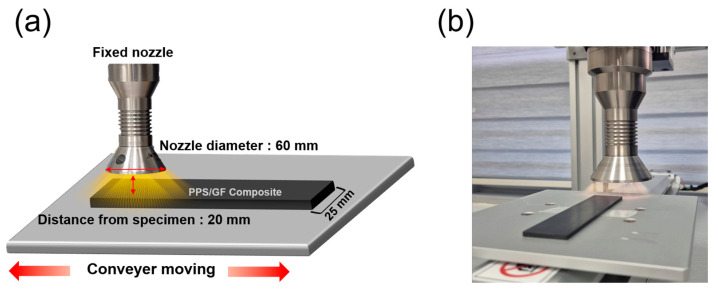
(**a**) Scheme of atmospheric-pressure plasma treatment for PPS/GF composites (Horizontal arrow: Nozzle diameter, Vertical arrow: Distance from specimen), (**b**) Photograph of PPS/GF composites under atmospheric-pressure plasma treatment.

The surfaces of the composites were treated using an atmospheric-pressure plasma apparatus (3D i REV, Applied Plasma Inc., Gumi, Republic of Korea) at an exposure power of 950 W. The plasma apparatus was supplied with compressed air at a pressure of 0.3 MPa and a frequency of 60 Hz. A nozzle with a diameter of 60 mm was fixed to the plasma apparatus, and the plate on which the composites were placed was moved back and forth at speeds of 1, 2, or 6 m/min. A slower plasma-treatment speed resulted in greater surface plasma irradiation. The area that can be treated at once is mainly determined by the width of the plasma nozzle because this width controls how wide the plasma spreads when it exits [24]. In this study, the nozzle width was 60 mm, while the specimen was only 25 mm wide, meaning that the entire surface could be moved back and forth to treat the entire surface at one time. As a result, all parts of the surface were equally exposed to the plasma, enabling uniform treatment. Figure 1 shows a schematic of the atmospheric-pressure plasma-treatment apparatus. Each specimen was treated with plasma once, and specimens without plasma treatment were used as control samples. Additionally, considering that the specimen length is 100 mm, even at the slowest treatment speed of 1 m/min, the plasma exposure takes only about 6 s. Thus, the total processing time remains under 10 s, demonstrating the high efficiency and speed of the proposed method.

### 2.3. Characterization

#### 2.3.1. Structural Analysis

The surface chemical composition of the PPS/GF composites was analyzed using XPS (Nexsa G2, Thermo Fisher, Waltham, MA, USA) with a monochromatic Al Kα X-ray source (1486.6 eV). For the XPS analysis, samples were prepared with dimensions of 25 mm × 25 mm × 3 mm. The oxygen functional groups introduced onto the PPS/GF composites were examined using attenuated total reflectance (ATR)-FT-IR spectroscopy (IRAffinity-1S, Shimadzu, Kyoto, Japan). The analysis was carried out in transmittance mode over the scanning range of 600–4000 cm^–1^.

#### 2.3.2. Morphological Analysis

The surface roughness change of the PPS/GF composites was analyzed using AFM (NX20, Park Systems, Suwon, Republic of Korea) in the non-contact mode. The surface morphologies of the PPS/GF composites were observed using field-emission (FE)-SEM (JSM-IT700HR, JEOL, Tokyo, Japan). The surface elemental distribution of the PPS/GF composites was analyzed using EDS data collected from the SEM. The specimens used for SEM analysis were prepared with dimensions of 12.5 mm × 12.5 mm × 3 mm using a diamond saw cutting machine.

#### 2.3.3. Wettability Analysis

The surface energies of the PPS/GF composites were determined by measuring the contact angles (Phoenix 300, SEO, Suwon, Republic of Korea) using the Owens–Wendt equation.

(1) $$\gamma_{S}=\gamma_{S}^{D}+\gamma_{S}^{P}$$

(2) $$\left(1+cos\theta \right)\gamma_{LV}=2{\left(\gamma_{S}^{D}\gamma_{LV}^{D}\right)}^{1/2}+2{\left(\gamma_{S}^{P}\gamma_{LV}^{P}\right)}^{1/2}$$

where $\gamma_{S}$ is the total surface energy of the PPS/GF composites; $\gamma_{LV}$ is the surface tension of the liquid; and $\gamma_{S}^{D}$ and $\gamma_{s}^{P}$ represent the dispersive and polar components, respectively. The contact angle (θ) measurements were performed using the sessile drop method with deionized (DI) water and diiodomethane. The surface tensions of the liquids are listed in Table 1 [25].

#### 2.3.4. Single-Lap Shear Testing

The adhesive used to evaluate the bonding strength was prepared using epoxy and a hardener provided by ESP Chem (Hwaseong, Republic of Korea). The epoxy and hardener were mixed in a weight ratio of 10:9, followed by degassing in a vacuum oven for 20 min. Subsequently, in accordance with the ISO 4587 standard, the adhesive was applied to the PPS/GF composites over an area of 12.5 mm × 25 mm [26]. Finally, plastic was attached to the grip to prevent specimen slippage and to ensure uniform load application during the bonding strength measurement.

**Figure 2:**
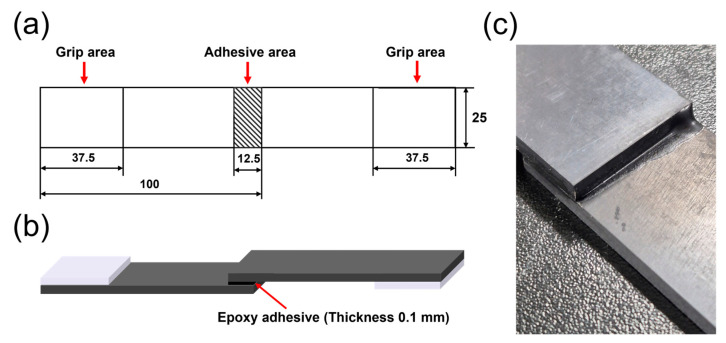
(**a**) Scheme of specimen dimensions in accordance of the ISO 4587 standards, (**b**) Schematic illustration of the single lap shear test specimen, and (**c**) Photograph the bonded region of the PPS/GF composites specimen for the single lap shear test.

The bonding strength between the PPS/GF composites and the epoxy adhesive was evaluated using a universal testing machine (AG-50kNX, Shimadzu, Kyoto, Japan) with a 50 kN load cell at a crosshead speed of 5 mm/min. The fractured surfaces after the single-lap shear test were observed using optical microscopy (OM; HVM0850D, Hana-vision, Seoul, Republic of Korea) to analyze the failure modes. Figure 2a shows the ISO 4587 standard dimensions of the specimen. Figure 2b,c show a schematic and a photograph of the PPS/GF composite specimen for the single-lap shear test.

## 3. Results

### 3.1. Structural Analysis of PPS/GF Composites

XPS is a widely recognized technique for detecting all elements, except hydrogen and helium, within ~10 nm of a polymer surface. The elemental composition of the samples was analyzed by deconvoluting the XPS spectra using CASA XPS software (version 2.3.26). Additionally, the chemical composition of the PPS/GF composite surface before and after plasma treatment was quantitatively characterized using XPS.

Figure S2 shows the XPS profiles of the PPS/GF composite surfaces obtained at varying plasma-treatment speeds. Table 2 shows the percentage of C, O, and S (at.%) and the oxygen ratio to carbon on the composites. The chemical changes were observed to be closely related to the plasma-treatment speed. As the treatment time increased, the concentration of oxygen atoms increased, corresponding to a lower treatment speed, whereas the carbon atom content decreased. The carbon content decreased from 86.51% in the untreated sample to 73.32% in the sample treated with plasma at a speed of 6 m/min. This reduction is attributed to plasma treatment, which removes organic pollutants from the surface and breaks or oxidizes carbon-based bonds. Conversely, the oxygen (O) ratio increased from 11.73% in the untreated specimen to 18.93% and 20.49% in the samples treated with plasma at speeds of 6 m/min and 1 m/min, respectively. As the plasma-treatment speed decreased, the plasma irradiation time on the surface increased, leading to greater incorporation of oxygen-containing functional groups. Meanwhile, the sulfur (S) ratio increased from 1.04% in the untreated specimen to 5.61% and 5.48% at 6 m/min and 2 m/min, respectively. This increase may be due to the removal of superficial low-molecular-weight contaminants or surface oxidation products during mild plasma exposure, thereby revealing more sulfur-containing groups of the PPS matrix. In addition, short-term plasma exposure may induce surface chain re-orientation, resulting in greater surface accessibility of sulfur atoms. However, at a treatment speed of 1 m/min, the sulfur content decreased to 2.11% owing to oxidation by the plasma treatment. At this lower speed, the plasma exposure was more intense and prolonged, potentially leading to excessive oxidation and surface etching. This could result in the formation of volatile sulfur compounds such as SO_2_ and SO_3_, which were subsequently desorbed and removed from the surface [27,28].

**Figure 3:**
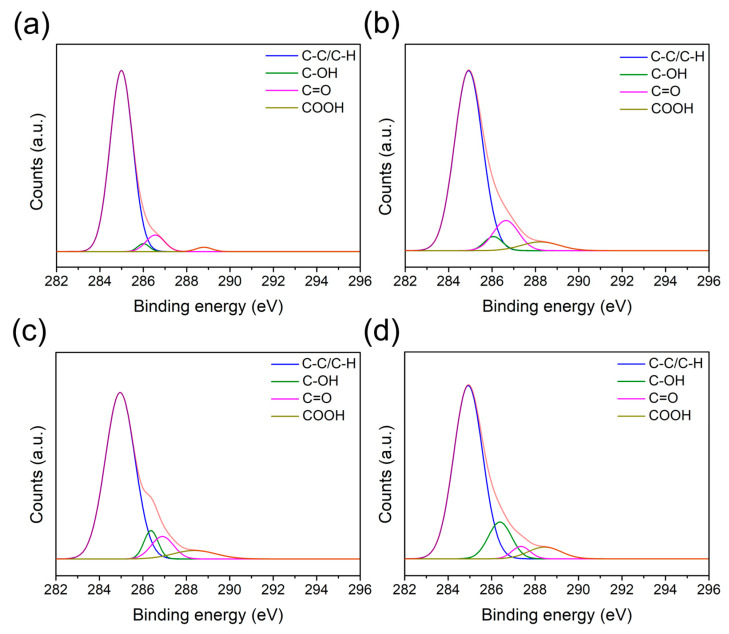
C1s spectra of PPS/GF composites at varying plasma-treatment speeds: (**a**) No plasma, (**b**) 6 m/min, (**c**) 2 m/min, and (**d**) 1 m/min. The orange curve represents the original C1s spectra before peak deconvolution.

The XPS C1s spectra of the PPS/GF composites at varying plasma-treatment speeds are shown in Figure 3 and summarized in Table 3. C1s peaks were deconvoluted into C–C (285 eV), C–OH (286.2 eV), C=O (286.9 eV), and COOH (288.7 eV) [29]. Comparing the control PPS/GF composites and the plasma-treated PPS/GF composites, the C–C/C–H ratio in the untreated sample was 89.67%, which decreased to 78.85%, 79.5%, and 77.45% at 6, 2, and 1 m/min, respectively. This reduction was likely due to the removal of organic contaminants from the surface and oxidation caused by the plasma treatment. On the contrary, the C–OH and COOH ratios increased from 2.05% (no plasma) to 4.02% (1 m/min plasma) and from 1.53% (no plasma) to 5.22% (1 m/min plasma), respectively. However, the C=O ratio, which increased from 6.74% to 11.91%, decreased to 8.11% at a speed of 2 m/min and to 3.46% at a speed of 1 m/min. This phenomenon is presumed to be due to the conversion of C=O to COOH, and this tendency becomes more pronounced as plasma irradiation increases.

**Figure 4:**
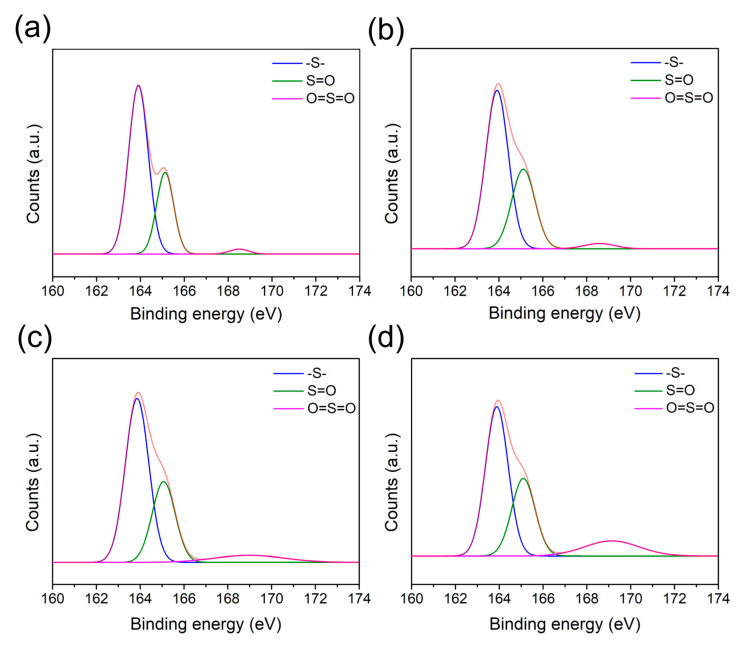
S2p spectra of PPS/GF composites at varying plasma-treatment speeds: (**a**) No plasma, (**b**) 6 m/min, (**c**) 2 m/min, and (**d**) 1 m/min. The orange curve represents the original S2p spectra before peak deconvolution.

The XPS S2p spectra of the PPS/GF composites at varying plasma-treatment speeds are shown in Figure 4 and summarized in Table 4. The S2p peaks were deconvoluted into –S– (163.9 eV), S=O (165.0 eV), and O=S=O (168.4 eV) peaks [27,30]. The –S– ratio of the control specimen, initially at 68.99%, decreased to 63.83%, 62.11%, and 56.28% at plasma-treatment speeds of 6, 2, and 1 m/min, respectively. This reduction was attributed to the etching effect induced by the plasma treatment and the oxidation of sulfur, leading to the formation of S=O and O=S=O bonds. At a speed of 6 m/min, the S=O and O=S=O ratios slightly increased from 29.09% (no plasma) to 33.29% and from 1.91% (no plasma) to 2.86%, respectively. However, at speeds of 2 and 1 m/min, the S=O ratios decreased to 30.01% and 30.07%, whereas the O=S=O ratios increased to 7.86% and 13.64%. These results indicate that the S=O bonds are gradually converted into O=S=O bonds, which are more stable [27].

**Figure 5:**
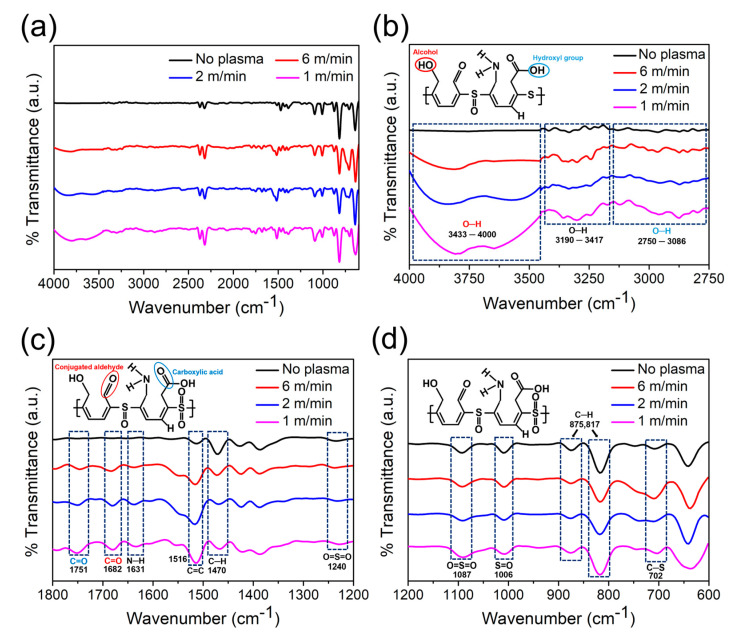
FT-IR spectra of the PPS/GF composites with various plasma treatment speeds (No plasma, 6 m/min, 2 m/min, 1 m/min), highlighting the formation and evolution of oxygen-containing functional groups: (**a**) Full range spectra (600–4000 cm^−1^), (**b**) O–H stretching region (2750–4000 cm^−1^), (**c**) carbonyl and aromatic C=C region (1200–1800 cm^−1^) and (**d**) Fingerprint region (600–1200 cm^−1^).

FT-IR spectroscopy is a widely used analytical technique for identifying functional groups and characterizing chemical bonds on polymer surfaces. ATR-FT-IR spectroscopy allows the direct analysis of the composite surface by measuring the IR absorption of the evanescent wave that penetrates a few micrometers into the material. The acquired spectra were analyzed to investigate the chemical modifications induced by the plasma treatment. The FT-IR spectra of the surface of the PPS/GF composites at varying plasma-treatment speeds are shown in Figure 5 and summarized in Table 5. Figure 5a shows the entire wavenumber range of 600–4000 cm^−1^. As shown in the 2750–4000 cm^−1^ region (Figure 5b), the plasma-treated specimens exhibit distinct peaks in the ranges of 3433–4000 cm^–1^, 3190–3417 cm^−1^, and 2750–3086 cm^−1^, indicating the formation of O–H and N–H groups. Specifically, the peaks in the 3190–3417 cm^−1^ range were attributed to C–OH, whereas those in the 2750–3086 cm^−1^ range were assigned to O–H and COOH groups. The intensities of these peaks increased as the plasma-treatment speed decreased, indicating enhanced surface functionalization. Figure 5c shows a zoom-in of the 1200–1800 cm^−1^ region. The peaks at 1682 cm^−1^ and 1751 cm^−1^ correspond to the C=O stretching vibrations of the conjugated aldehyde and carboxylic acids, respectively. Additionally, the peak at 1631 cm^−1^ is attributed to the N–H bending vibration. The presence of O–H, N–H, COOH, and C=O peaks indicate surface modification induced by plasma treatment. The peaks at 1470 cm^−1^ and 1516 cm^−1^ are related to the aromatic ring in the PPS molecule structure. The peak at 1516 cm^–1^ corresponds to the C=C stretching vibration of the aromatic ring, and its intensity increased as the surface organic contaminants were removed by plasma treatment. The peak at 1470 cm^−1^ exhibited weaker intensity as the plasma-treatment speed decreased, indicating the breaking of C–H bonds. The sulfone group exhibits two S=O stretching peaks, one of which appears at 1240 cm^−1^. Figure 5d presents a magnified view of the wavenumber range of 600–1200 cm^−1^, where the other sulfone peak can be observed at ~1087 cm^−1^. The peaks at 875 cm^−1^ and 817 cm^−1^ correspond to the out-of-plane C–H bending vibrations of the aromatic ring within the PPS structure, while the peak at 702 cm^–1^ represents the stretching vibration of the C–S bond [31,32].

### 3.2. Morphology of PPS/GF Composites

**Figure 6:**
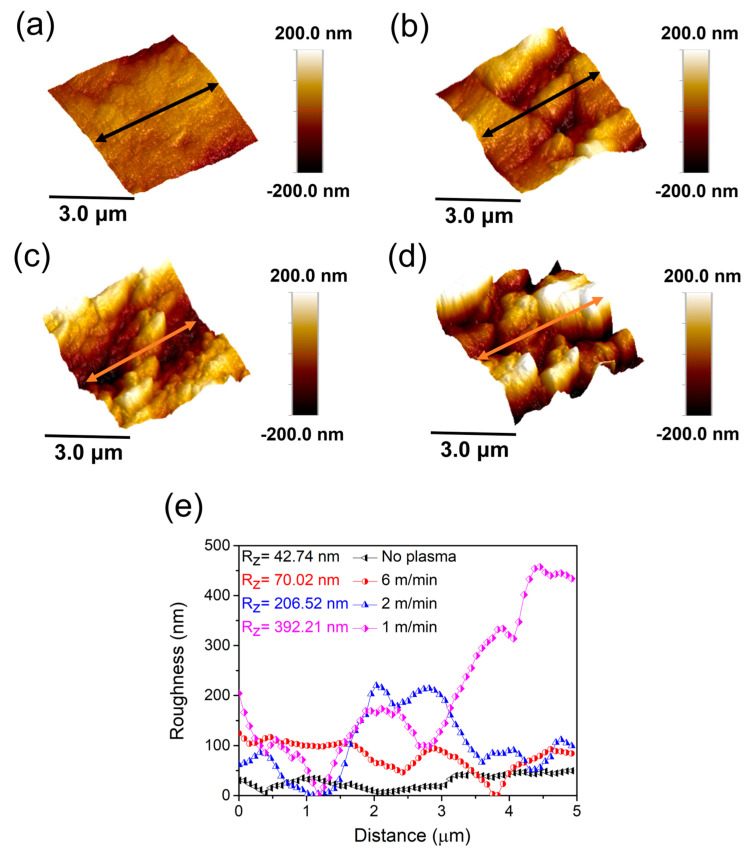
Surface roughness profile of PPS/GF composites at varying plasma-treatment speeds: (**a**) No plasma, (**b**) 6 m/min, (**c**) 2 m/min, and (**d**) 1 m/min. (**e**) Roughness profile of the arrow section along the longitudinal direction of the 5 μm scale.

The surface roughness profile and 10-point average roughness (R_z_) of the PPS/GF composites at varying plasma-treatment speeds are shown in Figure 6. At a plasma-treatment speed of 6 m/min, R_z_ increased by 64%, from 42.74 nm to 70.02 nm. This increase can be explained by the partial removal of surface contaminants and mild surface etching caused by reactive species in the plasma. At lower plasma-treatment speeds of 2 m/min and 1 m/min, the R_z_ significantly increased to 206.52 nm (+383%) and 392.21 nm (+817%), respectively, compared with the control sample. At lower treatment speeds, the longer exposure time allows the plasma to more strongly react with the surface. This causes more noticeable etching, forming uneven microstructures. These structures increase the surface area, improving mechanical interlocking with the epoxy adhesive.

**Figure 7:**
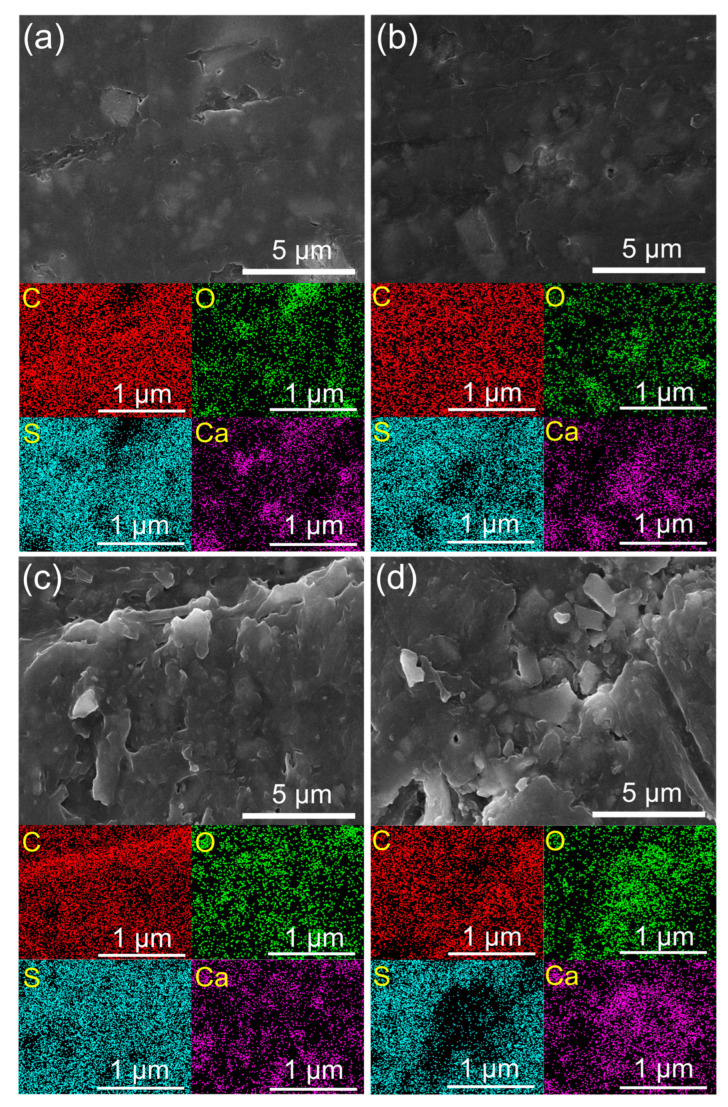
SEM images and EDS mapping of PPS/GF composites at varying plasma-treatment speeds: (**a**) No plasma, (**b**) 6 m/min, (**c**) 2 m/min, and (**d**) 1 m/min.

SEM images and EDS mapping of the PPS/GF composites at varying plasma-treatment speeds are shown in Figure 7. These images clearly illustrate the etching effects on the composite surface as a function of the plasma-treatment speed, confirming the trend of increasing surface roughness with prolonged plasma irradiation. Additionally, EDS analysis revealed that oxygen was concentrated in the plasma-treated regions, whereas the carbon and sulfur contents were partially reduced. This suggests that surface etching and oxidation occurred simultaneously, removing surface impurities while activating the surface chemically.

### 3.3. Wettability Analysis of PPS/GF Composites

**Figure 8:**
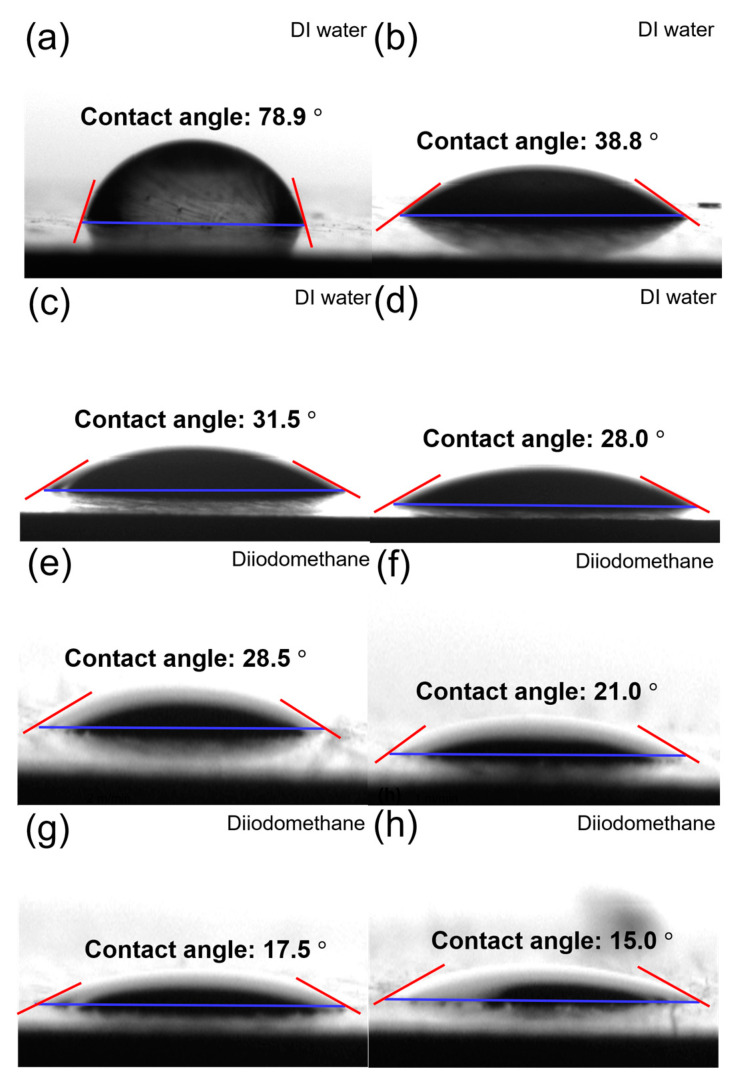
Contact angles measured using DI water and diiodomethane on the surface of the PPS/GF composites at varying plasma-treatment speeds: (**a**,**e**) No plasma, (**b**,**f**) 6 m/min, (**c**,**g**) 2 m/min, and (**d**,**h**) 1 m/min. (**a**–**d**) show DI water droplets, and (**e**–**h**) show diiodomethane droplets. Red lines indicate the fitted tangent at the droplet-solid interface used to calculate the contact angle, and blue lines represent the baseline of the solid surface.

Wettability describes how well a liquid spreads on a solid surface and plays a crucial role in bonding strength. Higher wettability increases the contact area between the adhesive and substrate, leading to stronger bonding. Wettability is typically evaluated using contact angle measurements, where a lower contact angle corresponds to better wettability. The contact angles—measured using diiodomethane (the dispersive component) and DI water (the polar component)—are shown in Figure 8. The water contact angle in the control sample was 78.9°, whereas at a plasma-treatment speed of 6 m/min, it decreased significantly to 31.5°, indicating a sharp increase in wettability. At speeds of 2 m/min and 1 m/min, the contact angles decreased further to 28.5° and 17.5°, respectively. This trend indicates that lower plasma-treatment speeds introduce a greater number of oxygen-containing functional groups onto the surface, thereby enhancing wettability. For diiodomethane, the contact angle of the control sample was 38.8°, which decreased to 28.0°, 21.0°, and 15.0° at 6, 2, and 1 m/min, respectively. Because diiodomethane contains only dispersive components, the reduction in contact angle can be attributed to the increase in surface roughness caused by the plasma treatment, which likely enhances wettability.

**Figure 9:**
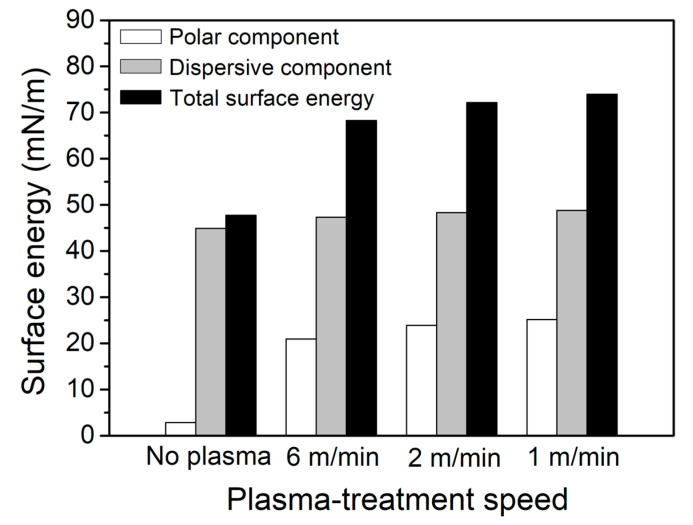
Variation in surface energy with plasma-treatment speed of the PPS/GF composites.

The surface energy components calculated by applying the Owens–Wendt equation are shown in Figure 9 and are summarized in Table 6 [33]. The total surface energy of the control sample was 47.7 mN/m, with 44.9 mN/m from the dispersive component and 2.8 mN/m from the polar component, indicating that the surface was predominantly dispersive and relatively inert. After plasma treatment at 6 m/min, the total surface energy increased by 43% to 68.2 mN/m, with 47.3 mN/m from the dispersive component and 20.9 mN/m from the polar component. While the dispersive component showed a slight increase, the polar component clearly increased, leading to an increase in the total surface energy. Similarly, at a speed of 2 m/min, the total surface energy reached 72.2 mN/m (48.3 mN/m dispersive, 23.9 mN/m polar), and at 1 m/min, it further increased to 74.0 mN/m (48.8 mN/m dispersive, 25.2 mN/m polar), representing increases of 51% and 55%, respectively. These results indicate that lower plasma-treatment speeds introduce a greater number of polar functional groups on the surface, leading to a significant increase in surface energy. This enhancement in surface energy is expected to improve the interfacial interactions between the PPS/GF composites and the epoxy adhesive, ultimately contributing to the improved bonding strength.

### 3.4. Adhesive Behavior of PPS/GF Composites

**Figure 10:**
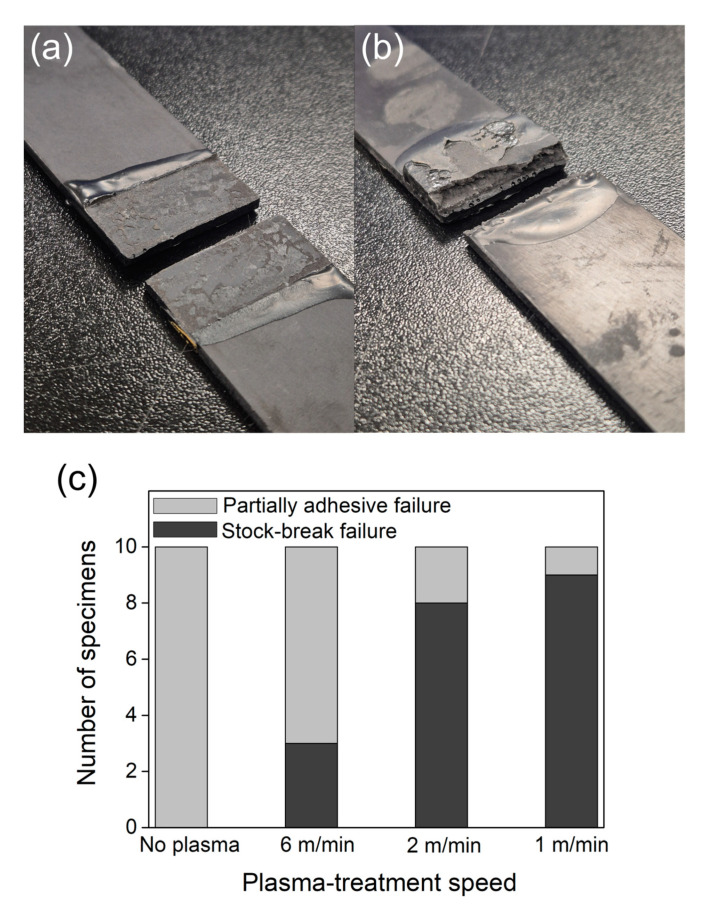
Representative failure morphologies and failure mode distribution according to plasma-treatment speed: (**a**) partial adhesive failure showing interfacial debonding, (**b**) stock-break failure indicating cohesive fracture within the substrate, and (**c**) failure mode distribution (partial adhesive vs. stock-break) across different plasma-treatment speeds.

Figure 10 illustrates the failure modes observed after single-lap shear testing and the trends in failure behavior according to the speed of plasma treatment. Figure 10a shows a partial adhesive failure mode, in which the epoxy adhesive delaminates from the composite surface due to insufficient interfacial bonding between the PPS/GF composites and the epoxy. The presence of residual epoxy on the composite surface is clearly visible in Figure S3, an optical microscope (OM) image. In contrast, Figure 10b presents a stock-break failure mode, where the failure occurs within the PPS/GF composite itself, indicating that the interfacial bonding strength exceeded the cohesive strength of the composite. Figure 10c summarizes the overall failure mode trends with respect to the plasma-treatment speed. All control samples exhibited partial adhesive failure. At a treatment speed of 6 m/min, some stock-break failures were observed. However, the failure mode was still predominantly adhesive. In contrast, most samples treated at 2 m/min and 1 m/min exhibited stock-break failure, indicating significantly improved adhesion. This change in failure mode is attributed to the increased incorporation of hydrophilic functional groups and the enhanced surface roughness caused by longer plasma exposure times, both of which contribute to stronger bonding with the epoxy [34].

**Figure 11:**
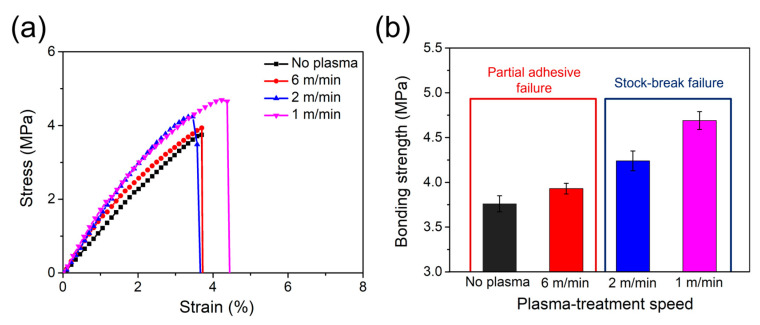
(**a**) Stress–strain curves and (**b**) bonding strength between PPS/GF composites and epoxy adhesives at varying plasma-treatment speeds.

The effect of plasma-treatment speed on mechanical performance is further demonstrated in Figure 11, which shows the stress–strain curve and bonding strengths, and in Table 7, which summarizes the numerical values. Compared with the control sample, a treatment speed of 6 m/min resulted in a 4.5% increase in bonding strength (from 3.76 MPa to 3.93 MPa). At speeds of 2 m/min and 1 m/min, the bonding strength further increased to 12.8% (4.24 MPa) and 24.8% (4.69 MPa), respectively, indicating a trend of lower plasma-treatment speeds leading to higher bonding strengths. Similarly, the elastic modulus increased compared with the control sample (113.76 MPa), reaching 146.52 MPa (+ 12.87%) at 6 m/min, 156.51 MPa (+13.76%) at 2 m/min, and 170.60 MPa (+ 15%) at 1 m/min. As discussed earlier, samples treated at speeds of 2 m/min and 1 m/min predominantly exhibited stock-break failure, which corresponds to their higher bonding strength and elastic modulus values. A lower speed of plasma treatment leads to improved interfacial adhesion, as evidenced by the transition from partial adhesive failure to stock-break failure and the enhancement in mechanical properties. Furthermore, as shown in Figure S4, there is a clear positive correlation between surface energy and bonding strength. The increase in surface energy, attributed to the introduction of polar functional groups, directly contributed to enhanced wettability and chemical interaction with the epoxy adhesive. Among all tested conditions, a plasma-treatment speed of 1 m/min was found to be the optimal, yielding the highest bonding strength.

## 4. Conclusions

In this study, the bonding strength between PPS/GF composites and epoxy was successfully enhanced through atmospheric-pressure plasma surface treatment. As the treatment speed decreased, the bonding strength increased, accompanied by a distinct transition in failure mode from partial adhesive failure to stock-break failure. This improvement is attributed to the increased introduction of oxygen-containing functional groups, such as hydroxyl (–OH) and carboxyl (–COOH) groups on the surface of the composites during prolonged plasma exposure, which promoted stronger chemical bonding with the epoxy adhesive. Additionally, the plasma treatment induced the surface etching of the PPS/GF composites, increasing surface roughness. This enhanced roughness expanded the contact area with the epoxy adhesive, thereby improving mechanical interlocking and further contributing to the enhanced bonding strength. At the optimized treatment speed of 1 m/min, the plasma treatment resulted in an 817% increase in surface roughness, a 55% increase in surface energy, and ultimately a 24.8% improvement in bonding strength compared with control samples. The findings of this study demonstrate that a simple and facile plasma treatment significantly improves adhesion performance, which plays a crucial role in the fabrication of capacitor housings for electric vehicles.

## Figures and Tables

**Table 1:** Surface tension values of DI water and diiodomethane.

Liquid	$\gamma_{LV}$ (mN/m)	$\gamma_{LV}^{D}$ (mN/m)	$\gamma_{LV}^{P}$ (mN/m)
DI water	72.8	51.0	21.8
Diiodomethane	50.8	50.8	0

**Table 2:** Percentage of elements on surface of PPS/GF composites at varying plasma-treatment speeds.

Plasma-Treatment Speed	Surface Elements (at.%)			
	C	O	S	O/C
No plasma	86.51	11.73	1.04	13.55
6 m/min	73.32	18.93	5.61	25.82
2 m/min	72.13	20.25	5.48	28.08
1 m/min	75.38	20.49	2.11	27.18

**Table 3:** Percentage of carbon bonds on surface of PPS/GF composites at varying plasma-treatment speeds.

Plasma-Treatment Speed	Carbon Bonds (at.%)			
	C–C/C–H	C–OH	C=O	COOH
No plasma	89.67	2.05	6.74	1.53
6 m/min	78.85	4.02	11.91	5.22
2 m/min	79.57	6.48	8.11	5.83
1 m/min	77.45	13.14	3.46	5.93

**Table 4:** Percentage of sulfur bonds on surface of PPS/GF composites at varying plasma-treatment speeds.

Plasma-Treatment Speed	Sulfur Bonds (at.%)		
	− S−	S=O	O=S=O
No plasma	68.99	29.09	1.91
6 m/min	63.83	33.29	2.86
2 m/min	62.11	30.01	7.86
1 m/min	56.28	30.07	13.64

**Table 5:** FT-IR spectral peak assignments of PPS/GF composites treated with plasma at atmospheric pressure.

Wavenumber (cm^–1^)	Assignment
3433–4000	O–H stretching of alcohol
3190–3417	O–H, N–H stretching
2750–3086	O–H stretching of hydroxyl group
1751	C=O stretching of carboxylic acid
1682	C=O stretching of conjugated aldehyde
1631	N–H bending of amine
1516	C=C stretching of aromatic ring
1470	C–H (in-plane) bending of aromatic ring
1240, 1087	S=O stretching of sulfone
1006	S=O stretching of sulfoxide
875, 817	C–H (out-of-plane) bending of aromatic ring
702	C–S–C stretching of PPS

**Table 6:** Polar, dispersive, and surface energies of the PPS/GF composites at varying plasma-treatment speeds.

Plasma-Treatment Speed	$\gamma_{S}^{D}$ (mN/m)	$\gamma_{S}^{P}$ (mN/m)	$\gamma_{S}$
No plasma	44.9	2.8	47.7
6 m/min	47.3	20.9	68.2
2 m/min	48.3	23.9	72.2
1 m/min	48.8	25.2	74.0

**Table 7:** Bonding strength and failure mode of PPS/GF composites at varying plasma-treatment speeds.

Plasma-Treatment Speed	Bonding Strength (MPa)	Elastic Modulus (MPa)	Failure Mode
No plasma	3.76 ± 0.09	113.76 ± 6.80	Partially adhesive
6 m/min	3.93 ± 0.06	146.52 ± 5.62	Partially adhesive
2 m/min	4.24 ± 0.11	156.51 ± 7.88	Stock-break
1 m/min	4.69 ± 0.10	170.60 ± 4.21	Stock-break

## Data Availability

All data used in this study appear in the submitted article.

## Supplementary Materials

The following supporting information can be downloaded at: https://www.mdpi.com/article/10.3390/polym17101344/s1. Figure S1: A schematic illustration of PPS/GF composites specimen preparation for single lap test. Figure S2: XPS spectra of PPS/GF composites with various plasma treatment speeds: (a) No plasma, (b) 6 m/min, (c) 2 m/min, and (d) 1 m/min. Figure S3: OM image of fractured surface showing partially adhesive failure at a plasma treatment speed of 6 m/min. Figure S4: Correlation between surface energy and bonding strength of PPS/GF composites treated by plasma irradiation with different treatment speeds. The red regression line illustrates the positive trend between increasing surface energy and corresponding bonding strength.
