# Supplementary material for: Improvement of Bonding Strength Between Polyphenylene Sulfide/Glass Fiber Composites and Epoxy via Atmospheric-Pressure Plasma Surface Treatment
Source: Polymers (Basel). 2025 May 14;17(10):1344. doi: 10.3390/polym17101344 (PMC12115051; doi:10.3390/polym17101344)
Supplement: Supplementary file 1 [file polymers-17-01344-s001.zip › polymers-3627775-supplementary.pdf]

Supplementary information

Improvement of Bonding Strength between PPS/GF Composites and Epoxy Through Atmospheric Pressure Plasma Surface Treatment

Hwan-Gi Do [1], Pyoung-Chan Lee [2,*] and Beom-Gon Cho [1,*]

[1] Department of Polymer Science and Engineering, Kumoh National Institute of Technology, 61 Daehak-ro, Gumi 39177, Gyeongbuk, Republic of Korea; 2025210703@kumoh.ac.kr

[2] Chassis & Materials Research Laboratory, Korea Automotive Technology Institute, 303 Pungse-ro,
Pungse-myeon, Dongnam-gu, Cheonan-si 31214, Chungcheongnam-do, Republic of Korea

* Correspondence: pclee@katech.re.kr (P.-C.L.); bgcho@kumoh.ac.kr (B.-G.C.); Tel.: +82-41-559-3144 (P.-C.L.); +82-54-478-7684 (B.-G.C.)

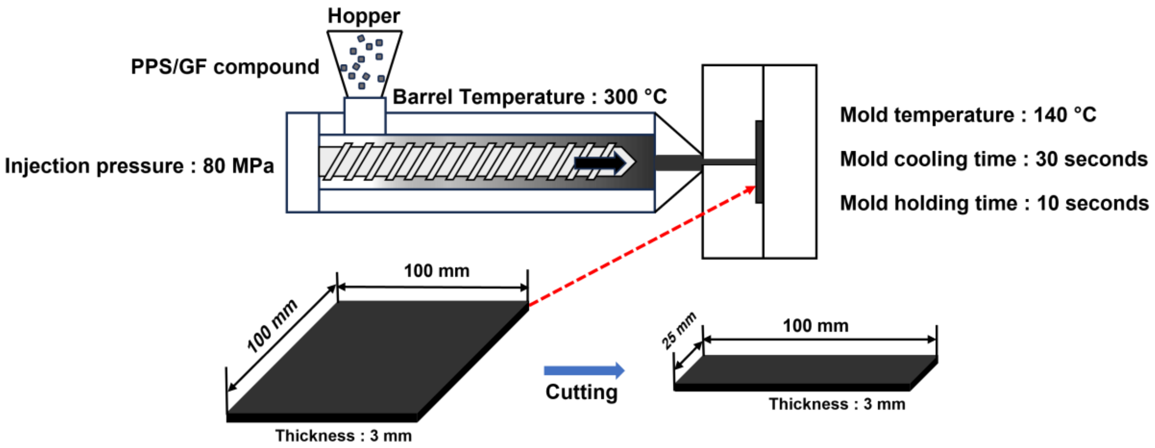

Figure S1. A schematic illustration of PPS/GF composites specimen preparation for single lap test.

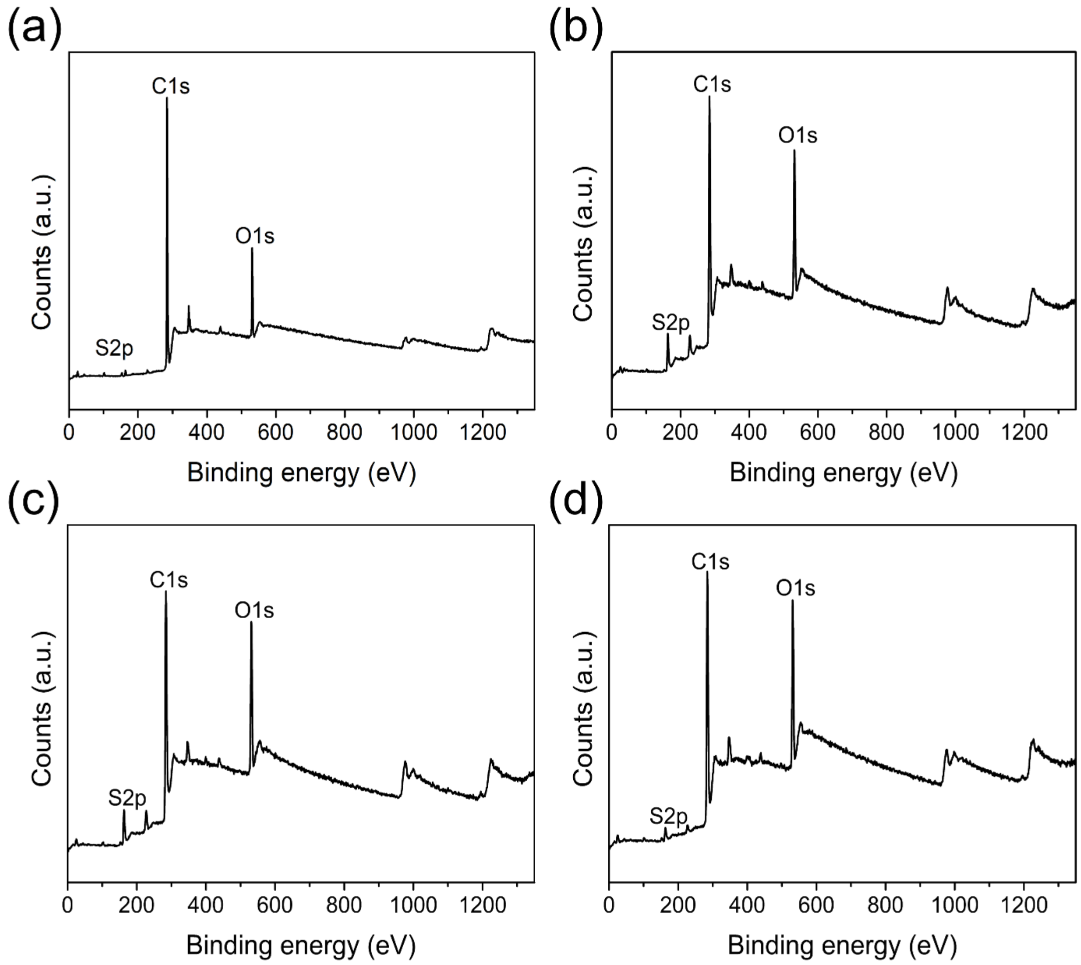

Figure S2. XPS spectra of PPS/GF composites with various plasma treatment speeds: (a) No plasma, (b) 6 m/min, (c) 2 m/min, and (d) 1 m/min.

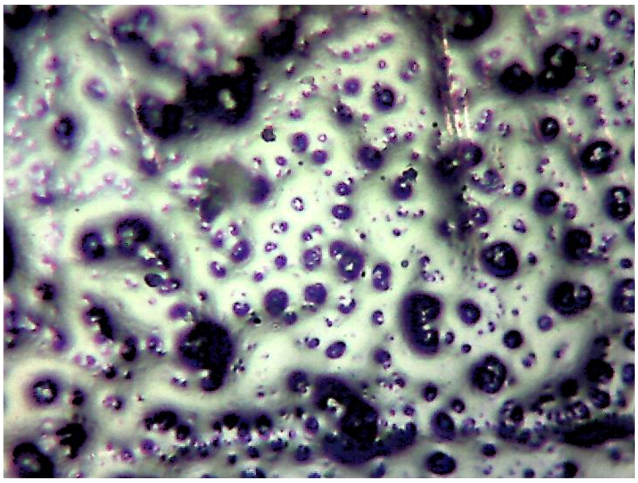

Figure S3. OM image of fractured surface showing partially adhesive failure at a plasma treatment speed of 6 m/min.

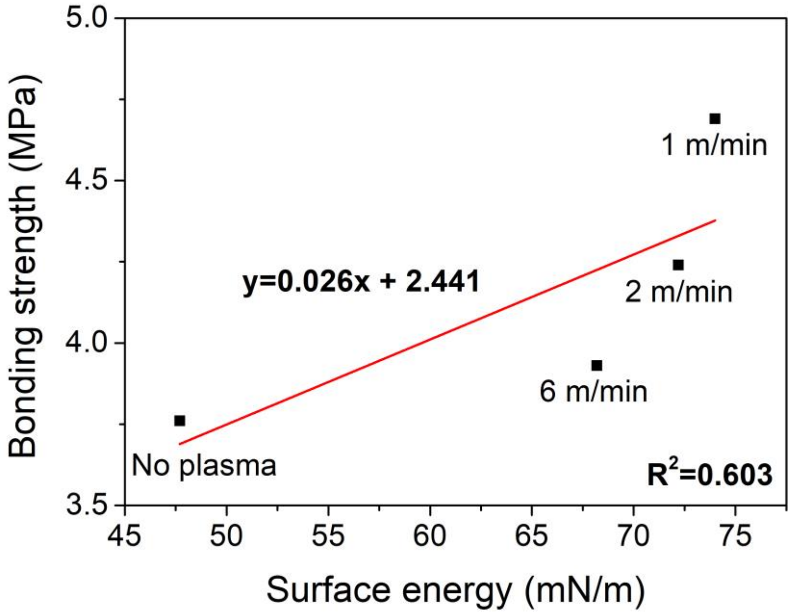

Figure S4. Correlation between surface energy and bonding strength of PPS/GF composites treated by plasma irradiation with different treatment speeds. The red regression line illustrates the positive trend between increasing surface energy and corresponding bonding strength.
